# Monstrosity

**Published:** 1852-03

**Authors:** 


*Monstrosity.*—M. Bèrard lately brought to the Academy of Medicine two lambs yeaned at the full period, which presented but one head. The neck was also single, but by compressing it, two vertebral columns might be felt; the sternum of each animal was soldered to the same bone in the other, and the upper part of the abdomen of each lamb was also connected. There were two distinct hearts, one being rudimentary, and situated anteriorly and to the right side, the other strong and large, posteriorly and to the left side. There were also two livers, one large on the right side, the other smaller to the left, the first of these organs receiving the umbilical vein, which came from the fœtus on the left. The disposition of parts within the skull was highly interesting, and was thus described:—Each spinal marrow enters the head by a distinct occipital hole, and the duality still persists within the head, which externally seems but single. There are two pontes Varolii, two calami scriptorii, there is a fourth ventricle on each side, and two cerebelli, which are connected in the mesian line. Higher up there is quite a collection of corpora quadrigemina, four of them in the centre quite in the normal state, and one situated on either side of this mass. The duality, however, ceased at that spot, for further on there is but one optic thalamus, one corpus striatum, and finally two hemispheres as might be seen in a slight fœtus.—*London Lancet.*

